# A clinical scoring system to predict the need for extensive resuscitation at birth in very low birth weight infants

**DOI:** 10.1186/s12887-019-1573-9

**Published:** 2019-06-14

**Authors:** Juyoung Lee, Jung Hyun Lee

**Affiliations:** 1 Department of Pediatrics, Bucheon St. Mary’s hospital, College of Medicine, The Catholic University of Korea, Seoul, South Korea; 2 Department of Pediatrics, St. Vincent’s Hospital, College of Medicine, The Catholic University of Korea, Jungbu-daero 93, Paldal-gu, Suwon-si, Gyeonggi-do Republic of Korea

**Keywords:** Neonate, Prediction model, Resuscitation, Very low birth weight

## Abstract

**Background:**

To analyze the risk factors for extensive cardiopulmonary resuscitation in the delivery room and develop a prediction model for outcomes in very low birth weight (VLBW) infants.

**Methods:**

The sample was 5298 VLBW infants registered in the Korean neonatal network database from 2013 to 2015. Univariate and multivariate analyses were used to analyze the risk factors for extensive resuscitation. In addition, a multivariable model predicting extensive resuscitation in VLBW infants was developed.

**Results:**

Univariate regression analysis of antenatal factors showed that lower gestational age, lower birth weight, birth weight less than third percentile, male sex, maternal hypertension, abnormal amniotic fluid volume, no antenatal steroid use, outborn, and chorioamnionitis were associated with extensive resuscitation at birth. Lower gestational age (25 to 27 gestational weeks, odds ratio [OR] and 95% confidence interval [CI]: 3.003 [1.977–4.562]; less than 25 gestational weeks, OR and 95% CI: 4.921 [2.926–8.276]), birth weight less than 1000 g (OR and 95% CI: 1.509 [1.013–2.246]), male sex (OR and 95% CI: 1.329 [1.002–1.761]), oligohydramnios (OR and 95% CI: 1.820 [1.286–2.575]), polyhydramnios (OR and 95% CI: 6.203 [3.185–12.081]), and no antenatal steroid use (OR and 95% CI: 2.164 [1.549–3.023]) were associated on multivariate regression analysis. The final prediction model for extensive resuscitation included gestational age, amniotic fluid, and antenatal steroid use. It presented a sensitivity of 0.795 and specificity of 0.575 in predicting extensive resuscitation at birth, corresponding to a score cut-off of 2. The area under the receiver operating characteristic curve was 0.738.

**Conclusions:**

Lower gestational age, abnormal amniotic fluid volume, and no use of antenatal steroid in VLBW infants are important predictors of extensive resuscitation in the delivery room.

## Background

Most newborn infants make the transition from intrauterine to extrauterine life without difficulty. About 10% need some assistance, and fewer than 1% require cardiac compression or medication in the delivery room [1]. However, among very low birth weight (VLBW) infants, approximately 90% need some kind of resuscitation and 4–10% require cardiac compression or medication [2–5].

The 2015 American Heart Association Guidelines Update for Cardiopulmonary Resuscitation and Emergency Cardiovascular Care recommend that every birth be attended by at least one person, and that additional personnel with full resuscitation skills should be immediately available for infants with significant perinatal risk factors that increase the likelihood of needing resuscitation [1]. Since most VLBW infants need positive pressure ventilation, two individuals usually attend these deliveries. In addition, when using extensive resuscitation, such as cardiac compression and epinephrine, at least three well-trained personnel, and needed resuscitation equipment and supplies are required.

Medical resources differ between countries and hospitals, as well as at different times of day and days of the week. Although the individual team members may have mastered the skills to resuscitate a newborn, they will not be able to use their skills optimally unless they work together as a team. Therefore, it is useful to be able to predict the need for resuscitation earlier than immediately prior to delivery, in order to save medical resources, especially in hospitals where they may be limited. Thus, our goal for this study was to establish a clinical prediction model, and to identify the antenatal risk factors associated with requiring extensive resuscitation in VLBW infants.

## Methods

### Study population

The Korean neonatal network (KNN) database is a national cohort registry of VLBW infants (< 1500 g) born in, or transferred within 28 days of birth to, one of the 66 neonatal intensive care units (NICUs) participating in the KNN. The database includes prospectively collected maternal data recorded at the time of birth, treatment process, and infant outcome data collected from birth until death, transfer, discharge, or 365 days after birth. Each KNN hospital’s institutional review board approved data collection for the KNN.

The present study included VLBW infants registered in the KNN database from 2013 to 2015. A VLBW infant who is born and admitted into NICU participating KNN or born in another hospital but transferred to the NICU of the KNN hospital within 28 days of birth was included. Infants with no record of resuscitation, premature rupture of membrane, amniotic fluid, or antenatal steroid use were excluded from analyses.

### Definitions of predictor and risk variables

VLBW was defined as birth weight < 1500 g. Birth weight < 10th or < 3rd percentile was determined based on sex-specific growth charts [6]. Maternal diabetes was based on diagnosis of gestational diabetes or overt diabetes during pregnancy. Maternal hypertension was based on any maternal diagnosis of pregnancy-induced hypertension or chronic hypertension in pregnancy. Oligohydramnios was defined as amniotic fluid index < 5. Polyhydramnios was defined as amniotic fluid index > 24. Antenatal steroid use was defined as any corticosteroid given to the mother during pregnancy to accelerate fetal lung maturity. Complete antenatal steroid status was based on two doses of betamethasone given at a 24-h interval, or four doses of dexamethasone at a 12-h interval, within 7 days before delivery; other administrations were defined as incomplete. Outborn was defined as born at another hospital and transferred to a hospital participating in the KNN. Chorioamnionitis was defined as the presence of acute inflammatory change in the amnion, chorion-decidua, umbilical cord, or chorionic plate based on histologic examination by a pathologist. Extensive resuscitation was defined as administration of chest compression, with or without administration of epinephrine, at birth in the delivery room.

### Statistical methods

Descriptive analyses were performed using Chi-square (χ^2^) or Fisher exact probability test for categorical variables, and independent t-test for continuous variables. To assess the association between extensive resuscitation and antenatal factors, logistic regression was performed.

To develop a prediction model based on available antenatal data, the data were randomly split into training (70%) and validation (30%) sets by statistical package. The data sets were comparable (data not shown). Using the training data set, a multivariable logistic regression model was constructed with extensive resuscitation as the outcome. Variables evaluated for inclusion in the prediction model were limited to those that could be measured before birth: maternal age, diabetes, hypertension, premature rupture of membrane, amniotic fluid volume, gestational age, and use of antenatal steroid. The final model was determined using backward elimination in which significant predictors remained in the model. A weighted scoring system was created using the square root of odds ratios (ORs) in the final model to the nearest integer. Receiver operator curve (ROC) analysis was used to determine the optimum cut-off score to predict extensive resuscitation; this was then applied in the validation set. Statistical analyses were conducted using SAS Version 9.4 (SAS Institute, Cary, NC) and a *P* value < 0.05 was considered statistically significant.

## Results

**Fig. 1:**
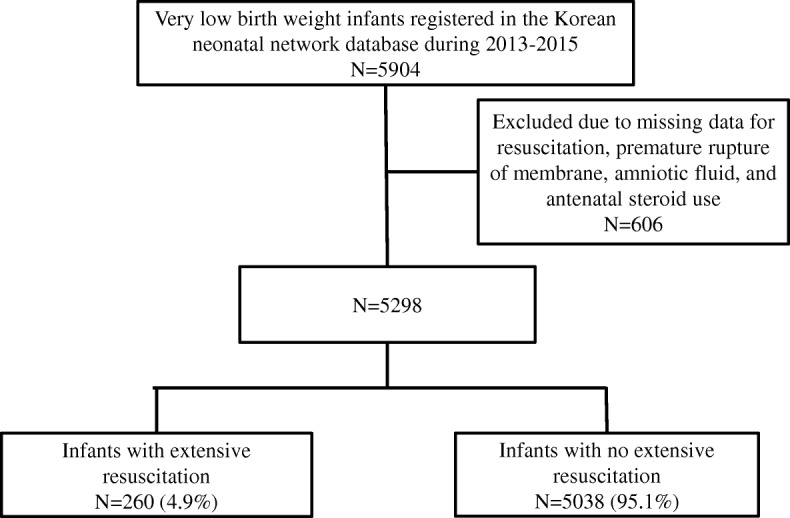
Flow chart of the study population

The study sample was 5298 VLBW infants (Fig. 1). A total of 5904 VLBW infants were registered in the KNN database during the study period. Among these, 15 infants had no recorded resuscitation, 44 infants were missing data for premature rupture of membrane, 521 infants were missing data for amniotic fluid, and 117 infants were missing data for antenatal steroid use. As some infants were missing more than one data, 606 infants were excluded and therefore, the final sample was 5298 VLBW infants. Extensive resuscitation occurred in 260 (4.9%) of these cases.

### Associations between antenatal factors and extensive resuscitation

Infants with lower gestational age, lower birth weight, lower 1- and 5-min Apgar scores, being outborn, and of male sex were associated with extensive resuscitation. Maternal characteristics of the infants who received extensive resuscitation included: hypertension, abnormal amniotic fluid volume (oligohydramnios or polyhydramnios), histologic chorioamnionitis, and no antenatal steroid use. Birth weight < 10th percentile, in vitro fertilization, multiple birth, maternal diabetes, premature rupture of membrane, and Cesarean section were not significantly related to the need for extensive resuscitation (Table 1).

**Table 1:** Maternal and infant characteristics

Characteristics	No extensive resuscitation (*n* = 5038)	Extensive resuscitation (*n* = 260)	*P* value
Gestational age (weeks)	28.96 ± 2.97	26.53 ± 2.52	<.001
Birth weight (g)	1087 ± 281	865 ± 290	<.001
Birth weight < 10th percentile^a^	1097 (21.9)	45 (17.6)	0.111
Birth weight < 3rd percentile^a^	529 (10.5)	16 (6.3)	0.029
Male	2520 (50.0)	150 (57.7)	0.016
Maternal age	32.78 ± 4.18	32.93 ± 4.20	0.572
In vitro fertilization	1104 (21.9)	46 (17.7)	0.107
Multiple births	1776 (35.3)	80 (30.8)	0.140
Maternal diabetes	411 (8.2)	17 (6.5)	0.350
Maternal hypertension	1076 (21.4)	36 (13.8)	0.004
Amniotic fluid			<.001
Normal	4255 (84.5)	189 (72.7)	
Oligohydramnios	702 (13.9)	55 (21.2)	
Polyhydramnios	81 (1.6)	16 (6.2)	
Premature rupture of membrane	1775 (35.2)	100 (38.5)	0.288
Antenatal steroid			<.001
None	1105 (21.9)	94 (36.2)	
Incomplete	1584 (31.4)	70 (26.9)	
Complete	2349 (46.6)	96 (36.9)	
Cesarean section	3880 (77.0)	195 (75.0)	0.452
Outborn	88 (1.7)	9 (3.5)	0.044
Chorioamnionitis^b^	1408 (33.0)	100 (44.2)	<.001
Apgar score			
1-min	4.75 ± 1.97	1.66 ± 1.38	<.001
5-min	6.95 ± 1.66	3.51 ± 2.11	<.001

^a^Could not be calculated for 25 infants (20 with no extensive resuscitation, 5 with extensive resuscitation) due to gestational age being out of range for the growth chart used

^b^Not collected for 804 infants (770 with no extensive resuscitation, 34 with extensive resuscitation)

Multivariate regression analysis of antenatal factors showed that lower gestational age (25 to 27 gestational weeks, OR and 95% confidence interval (CI): 3.003 [1.977–4.562]; less than 25 gestational weeks, OR and 95% CI: 4.921 [2.926–8.276]), birth weight less than 1000 (OR and 95% CI: 1.509 [1.013–2.246]), male sex (OR and 95% CI: 1.329 [1.002–1.761]), oligohydramnios (OR and 95% CI: 1.820 [1.286–2.575]), polyhydramnios (OR and 95% CI: 6.203 [3.185–12.081]), and no antenatal steroid use (OR and 95% CI: 2.164 [1.549–3.023]) were associated with extensive resuscitation at birth. Birth weight < 3rd percentile, maternal hypertension, outborn, and histologic chorioamnionitis were associated with the need of extensive resuscitation on univariate analysis, but not on multivariate analysis (Table 2).

**Table 2:** Regression analyses for antenatal factors predicting extensive resuscitation (*N* = 5298)

Antenatal factor	Univariate		Multivariate	
	OR (95% CI)	*P* value	OR (95% CI)	*P* value
Gestational age				
> 27 weeks	1.0 (Reference)		1.0 (Reference)	
25–27 weeks	3.521 (2.600–4.768)	<.001	3.003 (1.977–4.562)	<.001
< 25 weeks	7.510 (5.366–10.509)	<.001	4.921 (2.926–8.276)	<.001
Birth weight < 1000 g	3.490 (2.678–4.547)	<.001	1.509 (1.013–2.246)	0.043
Birth weight < 3rd percentile	0.568 (0.340–0.950)	0.031	0.836 (0.481–1.453)	0.525
Male	1.363 (1.059–1.753)	0.016	1.329 (1.002–1.761)	0.048
Maternal hypertension	0.592 (0.412–0.847)	0.004	1.047 (0.693–1.582)	0.826
Amniotic fluid				
Normal	1.0 (Reference)		1.0 (Reference)	
Oligohydramnios	1.764 (1.293–2.407)	<.001	1.820 (1.286–2.575)	0.01
Polyhydramnios	4.447 (2.551–7.752)	<.001	6.203 (3.185–12.081)	<.001
Antenatal steroid				
None	2.082 (1.552–2.791)	<.001	2.164 (1.549–3.023)	<.001
Incomplete	1.081 (0.789–1.481)	0.626	0.989 (0.702–1.395)	0.951
Complete	1.0 (Reference)		1.0 (Reference)	
Outborn	2.017 (1.004–4.052)	0.049	0.903 (0.209–3.902)	0.891
Chorioamnionitis	1.612 (1.230–2.112)	0.001	1.143 (0.851–1.536)	0.374

### Predictive model development

**Fig. 2:**
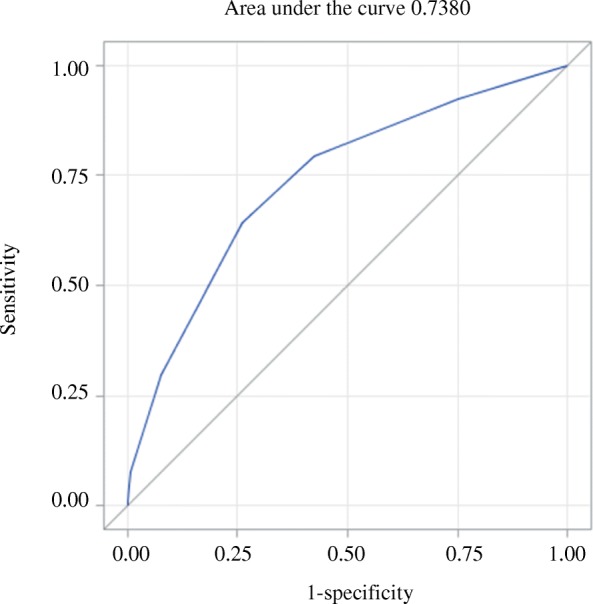
Receiver operating characteristic curve for the ability of the scoring model to predict extensive resuscitation, training set

We excluded sex, birth weight, and histologic chorioamnionitis, which cannot be clearly determined before labor. The final prediction model for extensive resuscitation included: gestational age, amniotic fluid, and antenatal steroid use. For the predictor variables, ORs were calculated and each variable was assigned a score, with the sums of the scores corresponding to an individual infant’s risk of requiring extensive resuscitation (Table 3). ROC analysis was used to determine the optimum cut-off value for the score in order to best predict extensive resuscitation. The highest sensitivity and specificity for the training data were 0.795 and 0.575, respectively (corresponding to a score cut-off of 2) (Table 4). At a score cut-off of 2, the positive predictive value (PPV) was 0.089 and negative predictive value (NPV) was 0.982 for the training set. The area under the ROC for the training set was 0.738 (Fig. 2). The validation data set model showed sensitivity 0.760, specificity 0.574, PPV 0.081, and NPV 0.980 at a score cut-off of 2 (Table 5). The area under the ROC for the validation set was 0.714.

**Table 3:** Final model for extensive resuscitation

Variable	Score assigned
Gestational age	
> 27 weeks	0
25–27 weeks	2
< 25 weeks	3
Antenatal steroid	
None or incomplete	1
Complete	0
Amniotic fluid	
Normal	0
Oligohydramnios	1
Polyhydramnios	2

**Table 4:** Estimated extensive resuscitation according to the risk score (training set)

Score	Sensitivity	Specificity	Positive predictive value	Negative predictive value
1	0.924 (0.886–0.962)	0.248 (0.233–0.262)	0.061 (0.052–0.069)	0.984 (0.976–0.992)
2	0.795 (0.736–0.853)	0.575 (0.558–0.591)	0.089 (0.076–0.103)	0.982 (0.976–0.987)
3	0.643 (0.574–0.712)	0.740 (0.726–0.755)	0.115 (0.096–0.135)	0.975 (0.969–0.981)
4 or more	0.297 (0.231–0.363)	0.925 (0.916–0.934)	0.172 (0.131–0.214)	0.962 (0.955–0.968)

**Table 5:** Estimated extensive resuscitation according to the risk score (validation set)

Score	Sensitivity	Specificity	Positive predictive value	Negative predictive value
1	0.933 (0.877–0.990)	0.256 (0.234–0.278)	0.059 (0.945–0.072)	0.987 (0.976–0.998)
2	0.760 (0.663–0.857)	0.574 (0.549–0.599)	0.081 (0.061–0.101)	0.980 (0.970–0.989)
3	0.587 (0.475–0.698)	0.723 (0.701–0.746)	0.095 (0.068–0.122)	0.973 (0.963–0.982)
4 or more	0.293 (0.190–0.396)	0.914 (0.900–0.928)	0.145 (0.089–0.201)	0.963 (0.953–0.973)

## Discussion

This is the first study to establish a prediction model for extensive delivery room resuscitation in VLBW infants. The model showed fair predictive accuracy.

Few previous studies have explored predictions of the need for neonatal resuscitation [7–10]. Aziz et al. found that maternal hypertension, maternal infection, multiple pregnancy, and oligohydramnios are independent risk factors for requiring positive pressure ventilation and/or endotracheal intubation. Their study included infants at 23 to 42 weeks’ gestational age and 9% were < 36 weeks [8]. A study by Afjeh et al. with a sample of infants with mean gestation of 37.4 weeks of whom 23.7% were preterm showed that low birth weight, meconium-stained fluid, and chorioamnionitis are independent risk factors for requiring endotracheal intubation [7]. However, it was unclear whether chorioamnionitis in their study was clinically and/or histologically based. In the current study, histologic chorioamnionitis was associated with extensive resuscitation in univariate analysis, but not in multivariate analysis. De Almeida et al. revealed that positive pressure ventilation in late preterm infants (34 to 36 weeks’ gestation) was associated with twin gestation, maternal hypertension, non-vertex presentation, Cesarean section, and lower gestational age [9]. Reis et al. proposed the use of a fuzzy expert system based on 61 risk situations to predict the need for positive pressure ventilation, endotracheal intubation, chest compression, and/or medications in the delivery room. Their sample was 10.2% preterm and 2.6% were < 34 weeks’ gestation [10].

Notwithstanding the previously established risk factors for neonatal resuscitation described herein, the goal of the present study was to establish early identification of risk factors in order to anticipate the need for personnel skilled in resuscitation. Thus, we included only those factors available in the KNN database that could be determined before birth. Each predictor variable has assigned a score, and the sums of the scores correspond to the infant’s risk of requiring extensive resuscitation. If the clinicians don’t have access to some of the variables, that pertinent score should be omitted. To date, there have been no data published to develop a clinical scoring system that can be individually used for the prediction of extensive delivery room resuscitation in VLBW infants.

Among the variables included in our prediction model, antenatal steroid use is the only modifiable risk factor. Several studies have shown that late preterm infants born to women who received antenatal steroid required less resuscitation at birth. A randomized trial showed that the betamethasone group required less resuscitation compared with the placebo group [11]. Even receiving a single dose of betamethasone led to less resuscitation [12]. A few studies have also shown that antenatal steroid is related to decreased extensive resuscitation [3]. In a population-based cohort study, infants who received chest compression and/or administration of epinephrine in the delivery room had received less antenatal steroid exposure compared with infants who did not receive extensive resuscitation, based on univariate analysis [13]. The current study supports the notion that antenatal steroid administration is significantly associated with decreased extensive resuscitation, based on univariate and multivariate regression analyses. Our final prediction model also included dose completion of antenatal steroid. The use of antenatal steroid improves lung function, with treated infants having higher Apgar scores and requiring less extensive resuscitation. Other factors may also contribute. The Brazilian Neonatal Research Network observed that antenatal corticosteroid-treated mothers had more prenatal medical visits compared with untreated mothers [14]. More prenatal care could result in improved pregnancy management, such as preventing preterm labor, thereby contributing to improved neonatal outcomes. In addition, there may have been urgent antenatal conditions (which are not provided in the KNN database) that disallowed enough time for steroid dose completion before delivery; such conditions may have independently affected neonates’ clinical status. In regression analysis, only no use of antenatal steroids had significant OR. However, prediction model could not be created when antenatal steroid was categorized as none and incomplete/complete. Additional multivariate regression analysis showed no or incomplete antenatal steroid has association with extensive resuscitation (OR and 95% CI: 1.54 [1.13–2.11]). Classifying antenatal steroid as none/incomplete and complete enabled us to develop a fair prediction model.

Abnormally decreased amniotic fluid volume may reflect fetal dysfunction that prevents normal urination, or it may represent a placental abnormality severe enough to impair perfusion [15]. Second-trimester rupture of the fetal membranes may also result in oligohydramnios [16, 17]. Few studies have investigated the correlation between oligohydramnios and resuscitation. Aziz et al. showed that oligohydramnios is a significant risk factor for positive pressure ventilation and/or endotracheal intubation [8]. Our analyses show that oligohydramnios is associated with extensive resuscitation in VLBW infants, and was one of the significant predictors of our model. Our data suggest that infants born to mothers with oligohydramnios who also have other unfavorable maternal and fetal conditions (e.g. fetal or placental insufficiency, premature rupture of membrane) have a worse prognosis at birth, and need more extensive resuscitation in the delivery room, compared with infants born to women with isolated oligohydramnios. This conclusion is consistent with the findings by Zhang et al. [18]

Common underlying causes of polyhydramnios include fetal congenital anomalies (approximately 15%) and diabetes (15–20%) [19]. Polyhydramnios is often a component of hydrops fetalis. Data regarding early neonatal complications from idiopathic polyhydramnios are conflicting. Some studies have found higher rate of newborn resuscitation with idiopathic polyhydramnios [20]. However, Panting-Kemp et al. showed that idiopathic polyhydramnios is unassociated with low 5-min Apgar score [21]. Our analyses support the notion that polyhydramnios is associated with extensive resuscitation in VLBW infants.

Our study aim was to develop a prediction model for extensive resuscitation using factors that can be clinically established before delivery. The variables we included were: gestational age, amniotic fluid, and antenatal steroid use. Our data suggest that the delivery by a woman with abnormal amniotic fluid volume, or in whom less antenatal steroid has been administered—despite gestational age > 27 weeks—should be prepared for extensive delivery room resuscitation. It should be attended by a team composed of at least three well-trained personnel with full resuscitation skills, and that equipment and supplies needed for extensive resuscitation should be prepared.

The present study was limited by only including risk factors available in the KNN database that could be determined before birth. Important variables that would allow assessment of the fetus, such as fetal heart rate, biophysical profile, fetal presentation, placenta abruptio, emergency Cesarean section, were not included in the KNN database. Male sex and extremely low birth weight infant were associated with extensive resuscitation at birth but were not used in the predictive model, because those factors cannot be clearly determined before labor. Another weakness of our predictive model is the low PPV. It results from the very low rate of extensive resuscitation. If a resuscitation team prepare for extensive resuscitation by using our model at a score cut-off of 2, only 8 out of 100 would be used. It could be safe for VLBW infants but not efficient, especially in hospitals with limited medical resources. Additional data and a larger sample, might improve the model for predicting extensive resuscitation in the delivery room.

The present study outlines factors that significantly increase the need for extensive resuscitation. These findings may be beneficial for developing strategies to anticipate circumstances that require more medical personnel and individuals with advanced neonatal resuscitation skills. Identifying risk factors and anticipating adequate levels of resuscitation that may be needed in the delivery room of a VLBW infant may better facilitate adequate preparation and prompt neonatal resuscitation, as well as target limited medical resources for those at the highest risk.

## Conclusions

This study is the first to propose a clinical scoring system to predict extensive delivery room resuscitation in VLBW infants. Lower gestational age, abnormal amniotic fluid volume, and less use of antenatal steroid in VLBW infants are important predictors of extensive resuscitation in the delivery room. However, further studies are required to improve the performance of the prediction model and increase sensitivity of extensive resuscitation in VLBW infants.

## Data Availability

The data that support the findings of this study are available from KNN network but restrictions apply to the availability of these data, which were used under license for the current study, and so are not publicly available.

## Abbreviations

CI: Confidence interval
KNN: Korean neonatal network
NICU: Neonatal intensive care unit
NPV: Negative predictive value
OR: Odds ratio
PPV: Positive predictive value
ROC: Receiver operator curve
VLBW: Very low birth weight
